# Domestic

**Published:** 1841-10-02

**Authors:** 


### MEDICAL EXAMINER.

DEVOTED TO MEDICINE, SURGERY, AND THE COLLATERAL SCIENCES.

No. 40.] PHILADELPHIA, SATURDAY, OCTOBER 2, 1841. [Vol. IV.

### DOMESTIC.

### HEALTH OF THE CITY

Interments *in the City and Liberties of Philadelphia, from the 18th to the 25th of September.*

| Diseases. | Adults. | Children. |
|---|---|---|
| Asthma, | 1 | 0 |
| Abscess of Lungs | 0 | 1 |
| Apoplexy, | 3 | 0 |
| Bowel Complaint, | 0 | 5 |
| Cancer of stomach | 1 | 0 |
| Casualty, | 0 | 1 |
| Congestion of Brain, | 0 | 1 |
| Colic, | 1 | 0 |
| Consumption of the lungs, | 10 | 2 |
| Convulsions, | 0 | 4 |
| —— Puerperal, | 1 | 0 |
| Chlorosis, | 0 | 1 |
| Cyanosis, | 0 | 1 |
| Diarrhœa, | 0 | 2 |
| Dropsy, head | 0 | 2 |
| Disease of the brain, | 1 | 0 |
| Dysentery, | 1 | 0 |
| Debility | 1 | 3 |
| Fever, | 1 | 0 |
| —— Remittent, | 0 | 1 |
| —— Bilious, | 1 | 0 |
| —— Typhus, | 2 | 1 |
| —— Typhoid, | 0 | 1 |
| —— Scarlet, | 0 | 3 |
| Hernia, | 2 | 0 |
| Inflammation of the Brain, | 0 | 3 |
| —— Bronchi, | 1 | [illegible] |
| —— Lungs, | 0 | 1 |
| *Carried forward,* | 27 | 33 |
| *Brought forward,* | 27 | 33 |
| —— Bowels, | 2 | 3 |
| —— Kidneys, | 1 | 0 |
| Intemperance, | 1 | 0 |
| Inanition, | 0 | 1 |
| Marasmus, | 0 | 6 |
| Neglect, | 0 | 2 |
| Old age, | 3 | 0 |
| Small pox, | 2 | 7 |
| Still-born, | 0 | 7 |
| Tabes Mesenterica, | 0 | 1 |
| Unknown, | 1 | 1 |
| Varioloid, | 0 | 1 |
| Total, 97— | 37 | 60 |

Of the above, there were under 1 year, 26

| | | | |
|---|---|---|---|
| From 1 | to | 2 | 12 |
| 2 | to | 5 | 10 |
| 5 | to | 10 | 4 |
| 10 | to | 15 | 0 |
| 15 | to | 20 | 8 |
| 20 | to | 30 | 9 |
| 30 | to | 40 | 7 |
| 40 | to | 50 | 3 |
| 50 | to | 60 | 6 |
| 60 | to | 70 | 6 |
| 70 | to | 80 | 4 |
| 80 | to | 90 | 2 |
| Total, | | | 97 |

Of the above there were 6 from the almshouse, 12 people of colour, and 2 from the country, which are included in the total amount.

